# Selenomethionine Improves Mitochondrial Function by Upregulating Mitochondrial Selenoprotein in a Model of Alzheimer’s Disease

**DOI:** 10.3389/fnagi.2021.750921

**Published:** 2021-10-12

**Authors:** Chen Chen, Yao Chen, Zhong-Hao Zhang, Shi-Zheng Jia, Yu-Bin Chen, Shao-Ling Huang, Xin-Wen Xu, Guo-Li Song

**Affiliations:** ^1^Shenzhen Key Laboratory of Marine Bioresources and Ecology, College of Life Sciences and Oceanography, Shenzhen University, Shenzhen, China; ^2^College of Physics and Optoelectronic Engineering, Shenzhen University, Shenzhen, China; ^3^Shenzhen Bay Laboratory, Shenzhen, China; ^4^Shenzhen-Hong Kong Institute of Brain Science-Shenzhen Fundamental Research Institutions, Shenzhen, China

**Keywords:** selenomethionine, mitochondrial selenoprotein, Alzheimer’s disease, mitochondria dysfunction and dementia, therapeutic effect and mechanism

## Abstract

Alzheimer’s disease (AD), the most common neurodegenerative disease in elderly humans, is pathologically characterized by amyloid plaques and neurofibrillary tangles. Mitochondrial dysfunction that occurs in the early stages of AD, which includes dysfunction in mitochondrial generation and energy metabolism, is considered to be closely associated with AD pathology. Selenomethionine (Se-Met) has been reported to improve cognitive impairment and reduce amyloid plaques and neurofibrillary tangles in 3xTg-AD mice. Whether Se-Met can regulate mitochondrial dysfunction in an AD model during this process remains unknown.In this study, the N2a-APP695-Swedish (N2aSW) cell and 8-month-old 3xTg-AD mice were treated with Se-Met *in vitro* and *in vivo*. Our study showed that the numbers of mitochondria were increased after treatment with Se-Met. Se-Met treatment also significantly increased the levels of NRF1 and Mfn2, and decreased those of OPA1 and Drp1. In addition, the mitochondrial membrane potential was significantly increased, while the ROS levels and apoptosis rate were significantly decreased, in cells after treatment with Se-Met. The levels of ATP, complex IV, and Cyt c and the activity of complex V were all significantly increased. Furthermore, the expression level of SELENO O was increased after Se-Met treatment. Thus, Se-Met can maintain mitochondrial dynamic balance, promote mitochondrial fusion or division, restore mitochondrial membrane potential, promote mitochondrial energy metabolism, inhibit intracellular ROS generation, and reduce apoptosis. These effects are most likely mediated *via* upregulation of SELENO O. In summary, Se-Met improves mitochondrial function by upregulating mitochondrial selenoprotein in these AD models.

## Introduction

Alzheimer’s disease (AD) is the most common form of dementia among elderly individuals and is an incurable neurodegenerative disease. Its two main pathological features are senile plaques formed by β-amyloid and neurofibrillary tangles caused by excessive amounts of phosphorylated microtubule-associated protein (tau) in the brain. These two pathologies can affect the normal functioning of mitochondria. Dysfunction of mitochondrial generation, energy metabolism, and other mitochondrial processes can promote the accumulation of Aβ and the hyperphosphorylation of Tau, which interact with each other and ultimately accelerate the pathological process of AD (Eckert et al., 2011). The brain is the most energy-consuming organ in the human body. Neurons have a unique oxidative ability and rely on sufficient oxygen and glucose for normal functioning (Khatri and Man, 2013). Mitochondria are the core organelles associated with energy metabolism in cells, so neurons are strongly dependent on mitochondria (Gonzalez-Lima et al., 2014). According to the mitochondrial cascade hypothesis, mitochondrial dysfunction precedes synaptic dysfunction, protein aggregation, and brain atrophy in AD (Hauptmann et al., 2006). A mitochondrial function may be influenced at three different levels: (1) structure and/or morphology (dynamics and quality control); (2) transport; and (3) biological energy metabolism and oxidative stress (Beal, 1995; Castellani et al., 2002; Cai and Tammineni, 2016).

Selenium is an essential biological trace element closely related to the maintenance of the normal functioning of the nervous system (Ying and Zhang, 2019). Inorganic selenium and organic selenium compounds (sodium selenite, sodium selenate, selenomethylselenocysteine, ebselen, etc.) have been reported to improve antioxidant ability, reduce the pathology of Aβ and tau, ameliorate synaptic deficits, and improve cognitive defects in AD mice (Van Rhijn et al., 1990; Van Eersel et al., 2010; Xie et al., 2017, 2018). Most selenium exists in the form of Se-Met in living organisms, and selenium mainly exerts its biological functions while incorporated in selenoproteins (Tapiero et al., 2003). Twenty-five selenoproteins have been found in the human brain (Reeves and Hoffmann, 2009). Only two of them are located in mitochondria: SELENO O and TRXR2 (Marciel and Hoffmann, 2017).

Since a mitochondrial dysfunction is an upstream event and pathological hallmark in AD pathology, protection of the normal functioning of mitochondria is essential for AD treatment. Long-term selenium deficiency increases the risks of neurodegenerative diseases such as AD (Loef et al., 2011). Previous studies in our laboratory have found that Se-Met treatment for 12 weeks significantly reduces the cognitive ability of 3xTg-AD mice, reduces Aβ deposition and tau and phosphorylated tau levels, and alleviates oxidative stress and synaptic damage (Zhang et al., 2016, 2017a,b). Thus, Se-Met has exhibited great potential for neuronal protection and maintenance of nervous system function. However, how Se-Met affects mitochondrial function and mitochondrial selenoprotein during this process remains unclear. Therefore, this study was conducted to further explore the effects of Se-Met on mitochondrial function, to investigate the regulatory role of mitochondrial selenoprotein both *in vivo* and *in vitro*, and to explore the protective mitochondrial mechanism involved in the treatment of AD with selenium.

## Materials and Methods

### Animals and Treatment

3xTg-AD mice were purchased from The Jackson Laboratory (JAX order number 3591206, Bar Harbor, ME, USA). These mice express the mutant PS1*M146V*, APP*swe*, and tau*P301L* transgenes and generally develop Aβ plaques and neurofibrillary tangles through a process representing the neuropathological process of AD. 3xTg-AD mice that were 8 months of age (*n* = 12; 6 males and 6 females) were treated with 6 μg/ml Se-Met (Sigma-Aldrich, USA) in drinking water for 12 weeks, while mice in the control group (*n* = 12; 6 males and 6 females) received double-distilled H_2_O (ddH_2_O). All diets were provided *ad libitum*.

The brains were removed from the AD mice immediately after sacrifice. The left hemisphere of each brain was processed for transmission electron microscopy (TEM). In parallel, the right hemisphere of each brain was further dissected into hippocampal and cortical samples, which were frozen in liquid nitrogen and stored at −80°C.

The surgical procedures and pre- and postoperative care of the animals conformed to the institutional guidelines regarding experimental animal use at Shenzhen University. All animal procedures were authorized by the Animal Ethical and Welfare Committee of Shenzhen University. All efforts were made to minimize animal suffering and to reduce the number of animals used.

### TEM

Fresh brain tissue was removed quickly and cut into cubes of approximately 2 mm^3^. Fixation was performed with 3% glutaraldehyde and 1.5% paraformaldehyde/0.1 M PBS (pH 7.2) at 4°C for more than 2 h. The tissues were rinsed three times with PBS, fixed with 1% osmic acid/1.5% potassium ferrocyanide at 4°C for 1.5 h, and washed with PBS three times. For dehydration, the tissues were soaked in 50% ethanol for 15 min, a 70% ethanol saturated uranium acetate dyeing solution at 4°C overnight, 90% ethanol for 15 min, a 90% ethanol and acetone mixture for 15 min, 90% acetone for 15 min, and anhydrous acetone for 15 min 3 times. The tissues were then incubated in an anhydrous acetone and epoxy resin 618 mixture for 1.5 h and in an embedding medium at 35°C for 3 h. Polymerization was carried out at 35°C for 12 h, 45°C for 12 h, and 60°C for 3 days. Then, 100 nm slices were sectioned with an ultramicrotome (Leica EM UC6). The slices were dyed with uranium acetate for 5 min and lead citrate for 15 min and then washed with double-distilled H_2_O. The images were photographed with a Philips EM208 transmission electron microscope.

### Immunoblot Analysis

In brief, brain tissues from transgenic mice were homogenized in nine volumes of RIPA buffer containing 1 mM PMSF supplemented with phosphatase inhibitors (Roche, Basel, Switzerland). Then, the homogenate samples were centrifuged at 13,000× *g* for 0.5 h at 4°C, and the supernatants were collected. The protein concentrations were determined using a bicinchoninic acid (BCA) assay kit (Sigma-Aldrich, USA). Twenty micrograms of protein were loaded per lane in a 10–15% SDS-polyacrylamide gel. After electrophoresis, the proteins in the gel were transferred onto 0.45 nm polyvinylidene difluoride membranes (Millipore, USA), which were incubated with primary antibodies diluted at 1:1,000 in TBS against the following proteins overnight at 4°C after blocking by 5% skim milk powder for at least 1 h: PGC-1α (Abcam, UK), DRP1 (Santa Cruz, USA), Mfn1, Mfn2 (Santa Cruz, USA), OPA1 (BD, USA), NRF1 and NRF2, Cyt c, GAPDH (Cell Signaling Technology, USA), COX IV (Abcam, USA), SELENO O (Abcam, USA), and TRXR2 (Cell Signaling Technology, USA). Subsequently, after being washed three times with Tris-buffered saline with Tween 20 (TBST), the membranes were incubated with anti-mouse and anti-rabbit secondary antibodies (Cell Signaling Technology, USA) in TBST for 1 h at 37°C. The blots were then developed using an enhanced chemiluminescence (ECL) kit (Amersham, Arlington Heights, IL) according to the manufacturer’s instructions and detected with a Tanon 5200 instrument (Tanon, China). The density of the bands was quantitated by scanning using densitometric software (ImageJ software). GAPDH was used as the internal control to normalize the protein loading.

### Cell Culture

#### Primary Neuron Culture

Primary neurons were isolated from the cortices of newborn AD mice and cultured at a certain concentration for various primary neuron cell experiments. Confocal plates were pretreated with polylysine (0.1 mg/ml, Sigma, USA). Cortices free of meninges were dissociated into 1 mm^3^ pieces in DMEM. The tissue was digested in papain for 0.5 h at 37°C. The tissue was then blown gently with a pipet in DMEM (supplemented with 0.2% glutamate, 1% rat serum, 2% B27, and 1% P/S). The supernatant containing single cells in suspension was collected. Suspend the cells to a density of 5 × 10^6^ cells/ml in the DMEM, and then the neurons were seeded in plates and incubated under 5% CO_2_ at 37°C. After 4–8 h, the DMEM was replaced with neurobasal medium supplemented with 0.2% glutamate, 2% B27, and 1% P/S for further culture. Half of the medium was replaced every 3 days. After 7 days of culture, the primary neurons were incubated with Se-Met (10 μM) for 24 h. After incubation, the neurons were dyed with JC-1 for 15 min for assessment of MMP.

#### Cell Line Culture

N2a-APP695-sw (N2a-SW), which is a mouse Neuro-2a cell line that stably expresses the APP695 mutations of a Swedish family, can express β-amyloid and is commonly used as an AD cell model. This cell line was provided by Professor Yunwu Zhang from Xiamen University and stored in our laboratory. The cells were cultured in complete medium (45% Opti-MEM, 45% DMEM, 10% FBS, G418) at 37°C under 5% CO_2_. The cells were collected in RIPA buffer containing 1 mM PMSF supplemented with phosphatase inhibitors (Roche, Basel, Switzerland). Then, the homogenate samples were centrifuged at 13,000× *g* for 0.5 h at 4°C, and the supernatants were collected for further western blotting.

### Flow Cytometry Analysis

#### MMP Detection

The MMP in N2a-SW cells was measured by flow cytometry with a JC-1 MMP detection kit. N2a-SW cells were collected and incubated with JC-1 solution for 20 min, then the culture medium was carefully removed and washed twice with JC-1 staining buffer (1×) for 2 min each time. The fluorescence intensity was measured in the FL-1 and FL-2 channels on a FACSCalibur flow cytometer (BD, USA; Perelman et al., 2012). The mean fluorescence intensity was recorded for 10,000 cells per group. Each sample was measured in triplicate, and three independent experiments were performed.

#### Apoptosis Level Detection

Apoptosis was detected by an Annexin V-FITC/PI apoptosis detect kit in N2a-SW cells. Cells were collected and washed twice by cold PBS, then resuspended in 1× Binding Buffer. Cells were incubated with FITC Annexin V and PI for 15 min at RT to avoid light, analyzed by flow cytometry within 1 h.

#### ROS Detection

The reactive oxygen species (ROS) levels were analyzed by a Reactive Oxygen Species Assay Kit (Beyotime Institute of Biotechnology, Shanghai, China). In brief, 1 × 10^6^ N2aSWs were collected in each group, washed by PBS, and incubated with fluorescent probes (DCFH-DA), for 20 min with 5% CO_2_ at 37°C. Then, cells were washed by a serum-free cell culture medium. Finally, ROS levels were detected by flow cytometry.

### Detection of Complex Activities in Mitochondria

The activities of mitochondrial complexes were detected by mitochondrial complex IV detection kit and mitochondrial complex V detection kit respectively (Solarbio life sciences, China). 5 × 10^6^ cells were collected and the activities of complex IV and complex V were detected, according to the instruction provided.

### Statistical Analysis

The data were analyzed using GraphPad Prism software. All data are expressed as the means ± standard errors of the means (SEMs). Statistical analyses were performed by unpaired *t*-test. Differences were considered significant at *p* < 0.05. Electronic laboratory notebook was not used.

## Results

### Se-Met Increased Mitochondrial Numbers and Alleviated Mitochondrial Vacuolation in N2a-SW Cells

**Figure 1:**
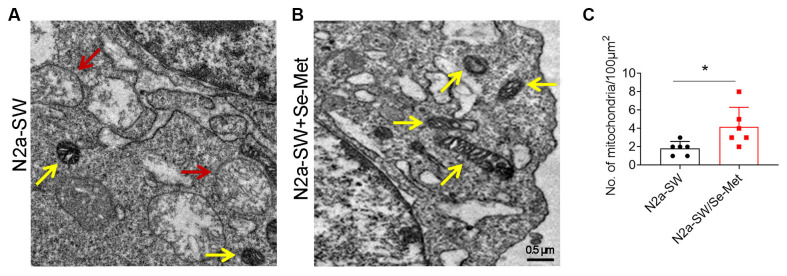
The mitochondrial morphology of N2a-SW cells was observed by TEM. **(A–C)** Representative images and corresponding quantification of mitochondria in N2a cell line following 10 μM Se-Met treatment. The red arrow represents the vacuolization of mitochondria. The yellow arrow represents normal mitochondria. 20,000×. Scale bars, 0.5 μm. *p < 0.05 vs. the control group. *n* = 6 slices.

After treatment with 10 μM Se-Met, the morphology of mitochondria in N2a-SW cells was observed by TEM (Figures 1A,B). The number of mitochondria in the control group was less than that in the Se-Met group. Moreover, a large number of mitochondria were vacuolated in the control group, which proved that Se-Met treatment reduced mitochondrial vacuolation and increased the number of mitochondria (Figure 1C).

### Se-Met Improved Mitogenesis by Upregulating the Expression of NRF1

**Figure 2:**
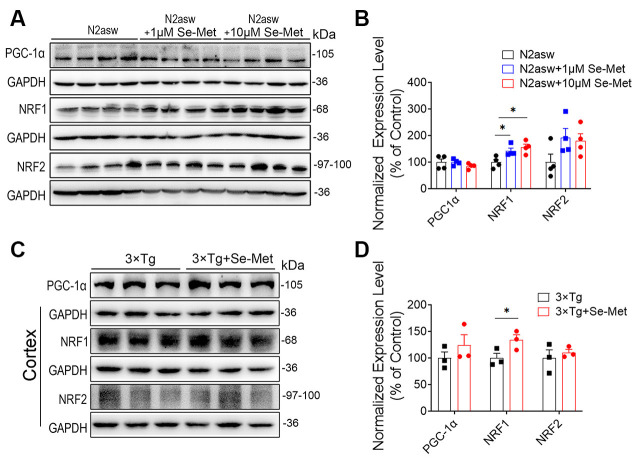
Se-Met improved mitogenesis in both N2a-SW cells and AD mice. **(A)** The levels of PGC1, NRF1, and NRF2 in N2a-SW cells were detected by western blot analysis. **(B)** Quantitative analysis of these proteins (*p < 0.05 vs. the control group. *n* = 4). **(C)** The levels of PGC1, NRF1, and NRF2 in the cortices of AD mice were detected by western blot analysis. **(D)** Quantitative analysis of these proteins (*p < 0.05 vs. AD mice. *n* = 3 mice).

To further explore the mechanism by which Se-Met treatment increased the number of mitochondria, mitogenesis in N2a-SW cells was evaluated. PGC-1, a subtype of PGC, exists mainly in skeletal muscle, heart, liver, and other mitochondria-rich tissues and participates in mitochondrial biosynthesis. NRF1 and NRF2 are important factors that promote mitochondrial biosynthesis. There were no significant differences in the expression levels of PGC-1 and NRF2 between the Se-Met-treated groups (both the 1 μM and 10 μM groups) and the control group. NRF1 protein levels were significantly increased in the 1 μM and 10 μM Se-Met groups (*p < 0.05, *n* = 4; Figures 2A,B). Similar phenomena were observed in the cortices of AD mice after Se-Met treatment, and the levels of NRF1 were significantly increased (*p < 0.05, *n* = 3; Figures 2C,D). These results indicated that Se-Met can promote mitochondrial biosynthesis in these AD models.

### Se-Met Promoted Mitochondrial Fusion and Inhibited Mitochondrial Division

**Figure 3:**
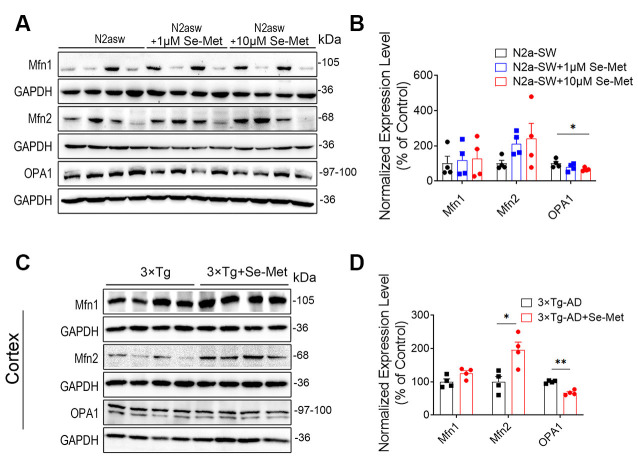
Se-Met improved mitochondrial fusion in both N2a-SW cells and AD mice. **(A)** The levels of Mfn1, Mfn2, and OPA1 in N2a-SW cells were detected by western blot analysis. **(B)** Quantitative analysis of these proteins (*p < 0.05 vs. the control group. *n* = 4). **(C)** The levels of Mfn1, Mfn2, and OPA1 in the cortices of AD mice were detected by western blot analyses. **(D)** Quantitative analysis of these proteins (*p < 0.05, **p < 0.01 vs. AD mice. *n* = 4 mice).

**Figure 4:**
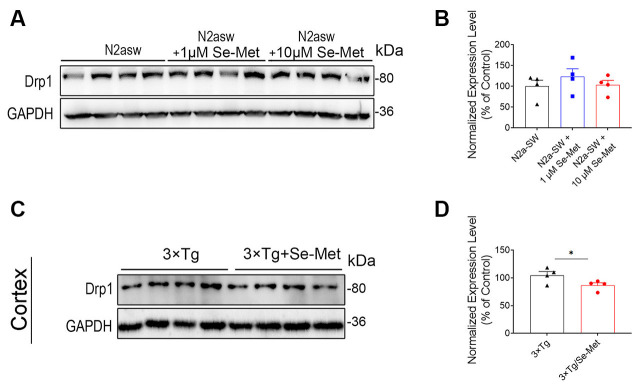
Se-Met inhibited mitochondrial fission in the cortices of 8-month-old AD mice. **(A–D)** The expression levels of Drp1 in N2a-SW cells **(A)** and in the cortices of AD mice **(C)** were detected by western blot analysis. **(B,D)** Quantitative analysis of the expression level of Drp1 (*p < 0.05, *n* = 4).

To explore the effects of Se-Met on mitochondrial dynamics, proteins related to mitochondrial fusion and division were detected. Mfn1 and Mfn2 regulate mitochondrial outer membrane fusion, and OPA1 participates in inner membrane fusion. Compared with the control cells, the Se-Met-treated N2a-SW cells exhibited no significant differences in the expression levels of Mfn1, Mfn2, and Drp1, but the cells treated with 10 μM Se-Met exhibited significantly decreased OPA1 levels (Figure 3). In addition, the expression levels of Mfn2 were significantly increased, while those of OPA1 were significantly decreased, in the cortices of AD mice after treatment (***p < 0.001, *n* = 4). The expression of Drp1, a dynamin-related protein that mediates mitochondrial division, was also decreased significantly in AD mice (*p < 0.05, *n* = 4; Figure 4).

### Se-Met Promoted Mitochondrial ATP Synthesis and Attenuated Mitochondrial Dysfunction

**Figure 5:**
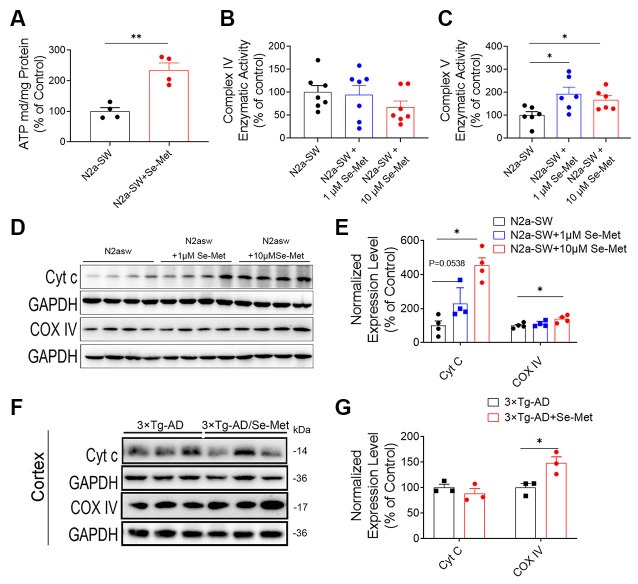
Se-Met increased ATP levels by promoting ATP synthesis. **(A)** ATP levels in the N2a-SW cell line treated with 10 μM Se-Met (**p < 0.01 vs. the control group, *n* = 4). **(B,C)** The enzymatic activity of complex IV **(B)** and complex V **(C)** was detected with a Micro Mitochondrial Respiratory Chain Activity Assay Kit (*p < 0.05 vs. the control group, *n* = 6). **(D–F)** The expression levels of Cyt c and complex IV in N2a-SW cells **(D)** and in the cortices of AD mice **(F)** were detected by western blot analysis. **(E–G)** Quantitative analysis of the expression levels of Cyt c and complex IV (*p < 0.05 vs. the control group. *n* = 4; *p < 0.05 vs. AD mice, *n* = 3 mice).

In general, decreases in MMP and decreases in ATP synthesis occur simultaneously during apoptosis. MMP and ATP synthesis were reported to decrease in the AD cells, indicating the existence of mitochondrial dysfunction (Frisard and Ravussin, 2006). Compared with the control group, the Se-Met group exhibited significantly higher levels of mitochondrial ATP synthesis (*p < 0.05, *n* = 6; Figure 5A). We further detected the levels of enzymes involved in the mitochondrial electron transport chain (ETC). The expression levels of complex IV significantly increased in both the N2a-SW cell line and 3xTg AD mice after treatment with Se-Met (Figures 5D–G), but there were no significant changes in enzymatic activity in the N2a-SW cell line (Figure 5B). However, the activity of complex V, an ATP synthetase, was significantly increased in both 1 μM and 10 μM Se-Met-treated N2a-SW cells (Figures 5C,D). In addition, the expression level of Cyt c was significantly increased after treatment with 10 μM Se-Met. These results indicate that Se-Met may promote ATP synthesis by attenuating mitochondrial dysfunction.

### Se-Met Prevented MMP Loss in an AD Cell Model

**Figure 6:**
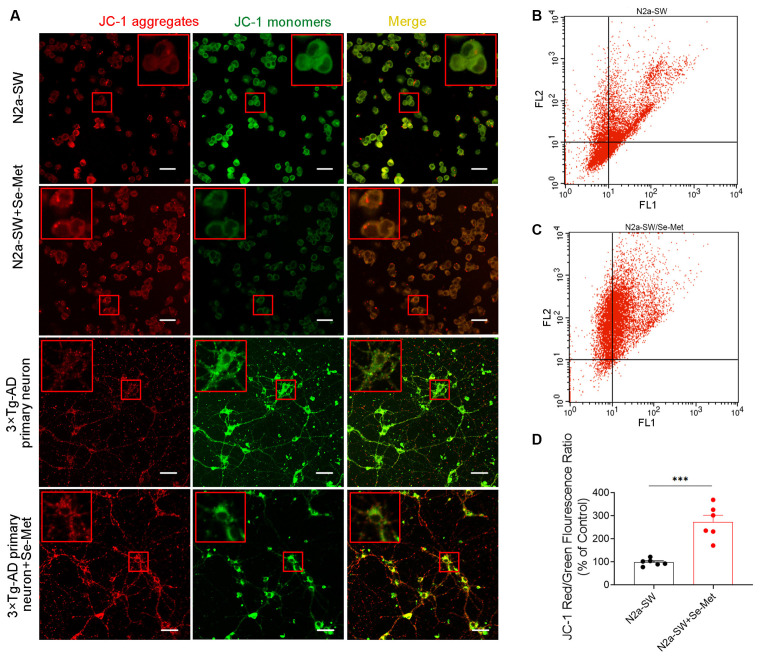
Effect of Se-Met on MMP as detected by JC-1 staining. **(A)** Confocal images showing the MMP measured using JC-1 as a probe. Scale bar, 10 μm. **(B–D)** Flow cytometry-based quantitative analysis of JC-1 in N2a-SW cells (***p < 0.001, *n* = 6).

To further explore the effect of Se-Met on apoptosis, MMP loss, an early event in apoptosis, was measured. The cyanine dye JC-1, which accumulates and fluoresces red in mitochondria in a potential-dependent manner, was used as an indicator. As shown in Figure 6, after treatment with 10 μM Se-Met for 24 h, the MMP was elevated, as indicated by increased red fluorescence and decreased green fluorescence of JC-1 in both N2a-SW cells and 3xTg-AD primary neurons. We further used flow cytometry to quantify the effect of Se-Met in N2a-SW cells. The ratio of red fluorescence to green fluorescence of JC-1 in N2a-SW cells was significantly increased after 10 μM Se-Met treatment (*p < 0.05, *n* = 4; Figure 6D). These results suggest that Se-Met effectively increases the MMP of AD neuronal cells.

### Se-Met Reduced ROS Levels and Apoptosis in the N2A-SW Cell Model

**Figure 7:**
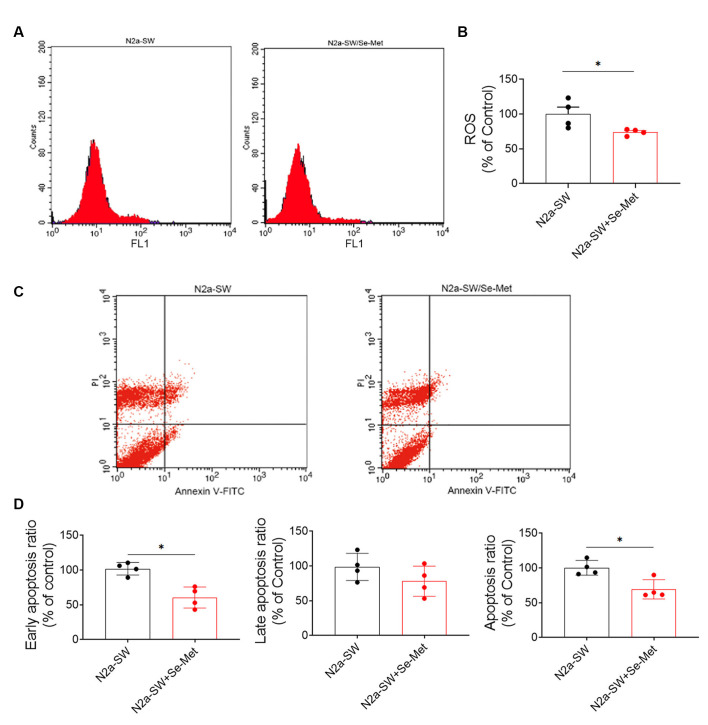
Se-Met attenuated cell damage and reduced apoptosis in the N2A-SW cell model. **(A)** ROS levels in N2a-SW cells treated with 10 μM Se-Met. **(B)** Quantitative analysis of the data in **(A)** (*p < 0.05 vs. the control group, *n* = 4). **(C)** Apoptosis was detected by flow cytometry. **(D)** Flow cytometry-mediated quantitative analysis of the early apoptosis, late apoptosis, and total apoptosis rates of the N2a-SW cell line (*p < 0.05 vs. the control group, *n* = 4).

Increased intracellular ROS levels are an important characteristic of neurons in AD and eventually lead to apoptosis, which is one of the main causes of cognitive decline. After treatment with 10 μM Se-Met for 24 h, the intracellular ROS levels in N2a-SW cells were detected by flow cytometry. As shown in Figures 7A,B, intracellular ROS levels were significantly lower in N2a-SW cells than in control cells (*p < 0.05, *n* = 6). We further used Annexin V-FITC/PI to detect apoptosis levels after Se-Met treatment. The early apoptosis rate (as shown in Figures 7C,D) (*p < 0.05, *n* = 6) and the total apoptosis rate were both decreased significantly (*p < 0.05, *n* = 6). These results indicate that Se-Met can reduce apoptosis by reducing ROS levels in the N2a-SW cell model.

### Se-Met Attenuated Mitochondrial Dysfunction by Upregulating SELENO O Both *In vivo* and *In vitro*

**Figure 8:**
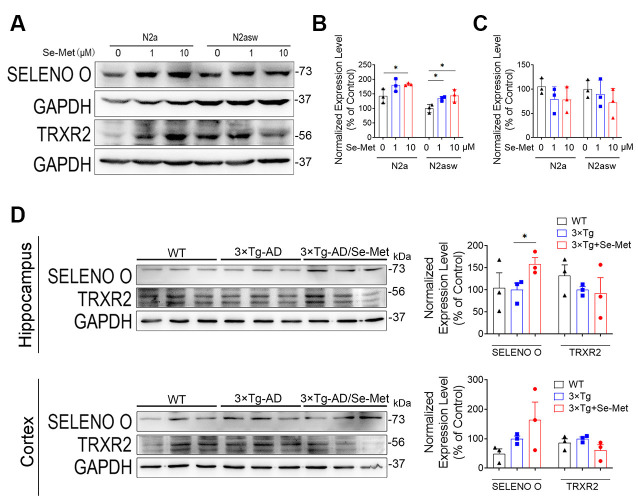
Se-Met may attenuate mitochondrial dysfunction by upregulating SELENO O both *in vivo* and *in vitro*. **(A–C)** Expression of SELENO O and TRXR2 in N2a and N2a-SW cells treated with 10 μM Se-Met. **(A)** The results of quantitative analyses of the expression levels of SELENO O **(B)** and TRXR2 **(C)** are shown (*p < 0.05 vs. the control group, *n* = 3). **(D)** Expression and quantified levels of SELENO O and TRXR2 in the hippocampi and cortices of 3xTg AD mice (*p < 0.05 vs. AD mice, *n* = 3 mice).

To explore the underlying mechanism of the effect of Se-Met on mitochondria, we detected two known selenoproteins located in mitochondria. Interestingly, we found that the levels of SELENO O were increased in both the N2a and N2a-SW cell lines and in the hippocampi of 3xTg AD mice after treatment with Se-Met (Figure 8). However, the expression levels of the other selenoprotein, TrxR2, showed no changes after Se-Met treatment. The restorative effect of Se-Met on mitochondrial function may be due to the upregulation of SELENO O.

## Discussion

AD is a complex, multifactorial, heterogeneous neurological disease characterized by memory loss and multiple cognitive impairments and is the most common form of dementia. Despite the increasing numbers of AD patients, few treatments are available to slow or halt the progression of this devastating disease (Lane et al., 2018). In AD, affected neurons exhibit mitochondrial dysfunction and bioenergetic deficits that occur early and promote disease-defining Aβ and Tau pathologies (Kerr et al., 2017). Selenium is now widely considered to be an essential microelement in the brain. Experimental evidence suggests that the selenium level in the brain decreases with age and that this decrease is associated with cognitive impairment in patients with AD (Cardoso et al., 2014). Se-Met is a selenium-substituted amino acid formed when the sulfur in methionine is replaced by selenium. It is an organic selenium compound with low toxicity and high bioavailability, the main form of selenium in living organisms, and an important chemical form of selenium absorbed by the body (Bodnar et al., 2012). Once ingested, Se-Met is absorbed *via* intestinal methionine transporters and enters the methionine pool in the body incorporated into their proteins at methionine positions (Combs and Combs, 1984; Wolffram et al., 1989; Daniels, 1996). Physiological cell selenium supply involves uptake of selenoprotein P (SELENOP), *via* transmembrane receptors. It is reported that SELENO P transports selenium across the blood-brain barrier and concentrates brain selenium in neurons *via* the Lrp8 receptor (Krol et al., 2012; Burk and Hill, 2015). In addition, Se-Met has been reported to alleviate cognitive deficits and oxidative damage in AD mice and to exhibit great potential for use in AD treatment (Zhang et al., 2016, 2017a,b). To explore the effects of Se-Met on mitochondrial function, the N2a-SW cell line and 3xTg-AD mice were used in this study.

Mitochondrial biogenesis plays an important role in maintaining healthy mitochondria in eukaryotic cells during the life cycle (Kotiadis et al., 2014). Once activated by either phosphorylation or de-acetylation, PGC-1α translocates from the cytoplasm into the nucleus to activate NRF1 and NRF2, and subsequently mitochondrial transcription factor A (Tfam) and mtDNA transcriptional regulators. The activation of this PGC-1α—NRF—Tfam pathway leads to the synthesis of mitochondrial DNA and proteins and the generation of new mitochondria (Li et al., 2017; Gureev et al., 2019; Venditti and Di Meo, 2020). Se-methylselenocysteine has been reported to promote mitochondrial biosynthesis in 14-month-old 3xTg-AD mice by increasing the expression levels of NRF1 and NRF2 (Xie et al., 2018). In this study, Se-Met had no significant effect on PGC1α, which may be related to the localization of PGC1α which needs to enter the nucleus for its biological function. The expression level of PGC we detected was in the whole fragments that may be the reason why there were no significant differences after Se-Met treatment in 3xTg-AD mice and N2a-SW cells. The NRF transcription factors are a subset of cap“n”collar transcriptional regulators and play distinct roles. NRF2 prefers binding to antioxidant-response elements flanked by GC-rich regions and NRF1 prefers AT-rich flanking regions (Liu et al., 2019; Ibrahim et al., 2020). Interestingly, the expression levels of NRF1 in 3xTg-AD mice and N2a-SW cells were significantly increased after Se-Met treatment, but not NRF2.

Energy generation depends on the ability of mitochondria to go through cycles of fission and fusion, collectively known as “mitochondrial dynamics” (Mishra and Chan, 2014). Under physiological conditions, mitochondria change from linear or tubular organelles to small, spherical organelles through rapid and reversible coordination of fission and fusion (van der Bliek et al., 2013). However, the balance can change to meet metabolic requirements, adapt to environmental stimuli or pathological conditions, or remove damaged organelles. Mitochondrial fusion is regulated by three GTPases, namely, Mfn1 and Mfn2 in the mitochondrial outer membrane and OPA1 in the intima (Sita et al., 2020). This process connects adjacent mitochondria together and merges their inner and outer membranes, ultimately forming a single mitochondrion. The fission process breaks one mitochondrion into two. Drp1 is transported to the outer mitochondrial membrane to start the oligomeric reaction, forming a large ring complex surrounding the future fission site along the outer surface of the mitochondrion (Ji et al., 2015). Studies have found that Drp1 expression is increased in the brains of AD patients, while Mfn1, Mfn2, and OPA1 expression is decreased (Baek et al., 2017). It has also been reported that reductions in Drp1 expression reduce Aβ production, reduce mitochondrial dysfunction, maintain mitochondrial dynamics, and enhance mitochondrial biogenesis and synaptic activity in APP mice (Manczak and Reddy, 2012; Manczak et al., 2016; Baek et al., 2017). In this study, Se-Met significantly increased the expression level of the mitochondrial fusion protein Mfn2 and decreased that of Drp1 in 3xTg-AD mice. These findings suggest that Se-Met promotes mitochondrial fusion, inhibits mitochondrial division, and maintains the dynamic balance of mitochondria. Recent studies have suggested that when OPA1 levels decrease, outer mitochondrial membranes can still fuse and promote fusion of the inner mitochondrial membranes through a series of processes (Ishihara et al., 2006). OPA1 exists in two types: long OPA1 regulates mitochondrial fusion, while short OPA1 can promote mitochondrial fission (Anand et al., 2014). It has been speculated that the significant OPA1 downregulation induced by Se-Met may be due to an effect on short OPA1 and its inhibition of mitochondrial fission. Therefore, Se-Met can promote mitochondrial fusion and inhibit mitochondrial division in these AD models.

Mitochondria produce ATP through OxPhos in the TCA cycle and ETC. The mitochondrial respiratory transport chain consists of four multisubunit complexes, I, II, III, and IV, and two electron carriers, ubiquinone/coenzyme Q and Cyt C. COX IV is the terminal electron acceptor of the mitochondrial ETC. The respiratory chain creates a proton gradient that drives ATP synthesis through ATP synthase, COX V. In this study, Se-Met treatment significantly increased the levels of ATP in N2a-SW cells. Moreover, Se-Met significantly increased COX IV and Cyt C levels, and COX V activity, indicating that Se-Met can promote COX IV-mediated conversion of O_2_ into water through oxidation of Cyt C and the ultimate generation of ATP. Se-Met may protect the key proteins that regulate mitochondrial energy metabolism and maintain normal physiological activities in this AD model.

Studies have shown that abnormalities in brain mitochondria lead to energy deficiency, increased ROS production, accelerated mitochondrial permeability transition pore opening, and decreased MMP in the 3xTg-AD mouse model. Moreover, a reduction in MMP is also a very early feature of apoptosis. In this study, it was found that Se-Met restored MMP in both 3xTg-AD primary neurons and N2a-SW cells and might maintain the normal physiological activity of mitochondria by reducing mitochondrial membrane damage. In OxPhos, 85–90% of intracellular oxygen is consumed by mitochondria, which produces harmful ROS. Due to inevitable electron leakage during electron transfer, superoxide anions are constantly produced in mitochondria despite the existence of efficient mitochondrial or cellular defense systems; this process leads to the production of 90% of endogenous ROS, which causes apoptosis. It has been reported that Aβ can enhance ROS production, and mitochondrial-derived ROS also trigger the formation of Aβ, increase BACE1 activity, accelerate mitochondrial dysfunction and Aβ production, and induce a vicious cycle (Curti et al., 1997). The results of this study showed that ROS and apoptosis levels were decreased significantly after treatment with Se-Met, suggesting that Se-Met can inhibit the production of ROS and reduce apoptosis.

TRXR2 and SELENO O are two selenoproteins located on mitochondria. Both of them have been reported to exhibit an oxidoreductase-regulated kinase function. TrxR2 is a key component of the mitochondrial thioredoxin system and is able to transfer electrons to peroxiredoxin 3 in a reaction mediated by thioredoxin 2 (Trx2; Forred et al., 2017). Inhibition of TrxR2 increases mitochondrial ROS concentrations and shifts the thiol redox state toward a more oxidized condition (Huang et al., 2015). SELENO O, the largest mammalian selenoprotein (with orthologs in a wide range of organisms), is a redox-active mitochondrial selenoprotein (Han et al., 2014). Its expression is affected little by selenium deficiency, suggesting that it is highly prioritized for selenium supply. In this study, we found that treatment with Se-Met did not significantly change the levels of TrxR2 in N2a cells, N2a-SW cells, and 3xTg AD mice, but it increased the levels of SELENO O. These results suggest that upregulation of SELENO O *via* Se-Met supplementation in AD models may be the key to improving mitochondrial function since selenium mainly exerts its effects through selenoproteins. Whether the effect of Se-Met on other mitochondrial functions is also directly mediated by selenoproteins O still requires further study.

## Innovation

This study uncovers that selenomethionine restores mitochondrial dynamic balance, promotes mitochondrial fusion or division, restores mitochondrial membrane potential, promotes mitochondrial energy metabolism, inhibits intracellular ROS generation and reduces apoptosis *via* upregulation of mitochondrial selenoprotein SELENO O. These findings suggest that mitochondrial selenoprotein may protects the mitochondria by affecting mitochondrial mechanisms involved in AD in the treatment of selenium.

## Conclusion

**Figure 9:**
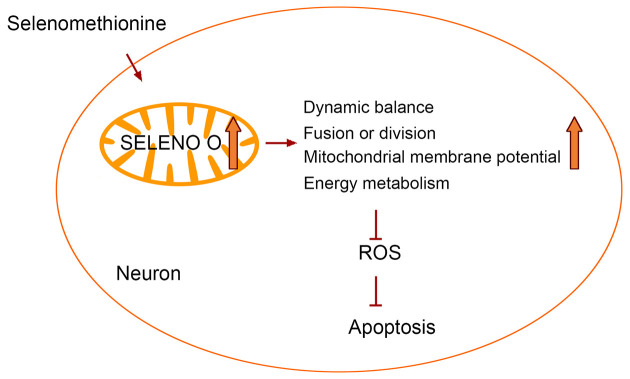
Selenomethionine improves mitochondrial function by upregulating mitochondrial selenoprotein in a model of Alzheimer’s disease.

In conclusion, our study shows that Se-Met can improve mitochondrial dysfunction, inhibiting intracellular ROS production, and reducing cell apoptosis, which is most likely exerted by upregulating mitochondria-resident selenoprotein SELENO O in AD cells. The study provides a novel and pleiotropic mechanism behind the therapeutic effect of Se-Met in AD (Figure 9).

## Data Availability Statement

The original contributions presented in the study are included in the article, further inquiries can be directed to the corresponding author.

## Ethics Statement

The animal study was reviewed and approved by the Animal Ethical and Welfare Committee of Shenzhen University (Permit Number: AEWC-20140615-002).

## Author Contributions

G-LS conceived the study, participated in the analysis and interpretation of data, drafted the manuscript, and revised it critically for intellectual content. CC participated in data generation, analysis and interpretation and drafted the manuscript, and revised it critically for intellectual content. YC participated in data generation and analysis. Z-HZ, S-ZJ, Y-BC, S-LH, and X-WX participated in the data generation and provided technical support. G-LS is the guarantor of this work and, as such, had full access to all the data in the study and takes responsibility for the integrity of the data and the accuracy of the data analysis. All authors contributed to the article and approved the submitted version.

## Conflict of Interest

The authors declare that the research was conducted in the absence of any commercial or financial relationships that could be construed as a potential conflict of interest.

## Publisher’s Note

All claims expressed in this article are solely those of the authors and do not necessarily represent those of their affiliated organizations, or those of the publisher, the editors and the reviewers. Any product that may be evaluated in this article, or claim that may be made by its manufacturer, is not guaranteed or endorsed by the publisher.

## Abbreviations

3xTg: AD mice-triple transgenic Alzheimer’s disease mice
AD: Alzheimer’s disease
ATP: adenosine triphosphate
COX V: complex V
Cyt c: cytochrome c
Drp1: dynamin-relatedprotein 1
ETC: electron transport chain
Mfn2: mitofusin 2
MMP: mitochondrial membrane potential
mtDNA: mitochondrial DNA
N2aSW: N2a-APP695-Swedish
NRF: nuclear respiratory factor; Mitochondrial transcription factor A (Tfam)
OPA1: optic Atrophy 1
OxPhos: oxidative phosphorylation
PGC-1: peroxisome proliferator-activated receptorγcoactivator-1
SELENO O: selenoprotien O
SELENO P: selenoprotien P
Se-Met: Selenomethionine
TRXR2: tioredoxin reductase 2.
