# Corrigendum: Selenomethionine Improves Mitochondrial Function by Upregulating Mitochondrial Selenoprotein in a Model of Alzheimer's Disease

**DOI:** 10.3389/fnagi.2021.815251

**Published:** 2021-12-20

**Authors:** Chen Chen, Yao Chen, Zhong-Hao Zhang, Shi-Zheng Jia, Yu-Bin Chen, Shao-Ling Huang, Xin-Wen Xu, Guo-Li Song

**Affiliations:** ^1^Shenzhen Key Laboratory of Marine Bioresources and Ecology, College of Life Sciences and Oceanography, Shenzhen University, Shenzhen, China; ^2^College of Physics and Optoelectronic Engineering, Shenzhen University, Shenzhen, China; ^3^Shenzhen Bay Laboratory, Shenzhen, China; ^4^Shenzhen-Hong Kong Institute of Brain Science-Shenzhen Fundamental Research Institutions, Shenzhen, China

**Keywords:** selenomethionine, mitochondrial selenoprotein, Alzheimer's disease, mitochondria dysfunction and dementia, therapeutic effect and mechanism

In the published article, there was an error in affiliation 1. Instead of “College of Life Sciences and Oceanography, Shenzhen University, Shenzhen, China,” it should be “Shenzhen Key Laboratory of Marine Bioresources and Ecology, College of Life Sciences and Oceanography, Shenzhen University, Shenzhen, China.”

In the published article, there was an error regarding the affiliation for Chen Chen. As well as having affiliation 1, they should also have “College of Physics and Optoelectronic Engineering, Shenzhen University, Shenzhen, China.”

There was another error regarding the affiliation for Guo-Li Song. As well as having affiliation 1, they should have “Shenzhen Bay Laboratory, Shenzhen, China” and “Shenzhen-Hong Kong Institute of Brain Science-Shenzhen Fundamental Research Institutions, Shenzhen, China.”

The authors apologize for this error and state that this does not change the scientific conclusions of the article in any way. The original article has been updated.

## Publisher's Note

All claims expressed in this article are solely those of the authors and do not necessarily represent those of their affiliated organizations, or those of the publisher, the editors and the reviewers. Any product that may be evaluated in this article, or claim that may be made by its manufacturer, is not guaranteed or endorsed by the publisher.

